# Acceptance of COVID-19 vaccination and its determinants among Lebanese dentists: a cross-sectional study

**DOI:** 10.1186/s12903-021-01831-6

**Published:** 2021-09-29

**Authors:** Lara Nasr, Nadine Saleh, Mira Hleyhel, Abbass El-Outa, Ziad Noujeim

**Affiliations:** 1 Department of Epidemiology and Biostatistics, Faculty of Public Health, Lebanese University, Fanar, Lebanon; 2 Department of Emergency Medicine, American University of Beirut Medical Center, Beirut, Lebanon; 3 Department of Oral and Maxillofacial Surgery, Faculty of Dental Medicine, Lebanese University, Hadat, Lebanon

**Keywords:** COVID-19 vaccines, SARS-CoV-2, Vaccination, Dentists

## Abstract

**Background:**

Dentists are at high risk of exposure to occupational Coronavirus Disease 2019 (COVID-19). Since vaccination is crucial to control COVID-19 pandemic, we aimed to assess COVID-19 vaccination acceptance and its determinants among Lebanese practicing dentists.

**Methods:**

A cross-sectional online study was conducted between February 15 and 22, 2021, among dentists practicing in Lebanon. Prevalence of COVID-19 vaccine acceptance was estimated. A multivariable modified Poisson regression model was used to explore determinants of COVID-19 vaccine acceptance.

**Results:**

In total, 86% of participants were willing to receive or have already received a COVID-19 vaccine. Having received the influenza vaccine during the COVID-19 pandemic was linked to a 12% increase in the COVID-19 vaccination acceptance rate. In addition, participants having moderate and high COVID-19 vaccination knowledge levels were more likely to accept receiving the vaccine, and participants whose fear of COVID-19 level was high were more likely to accept receiving the vaccine compared to those having a low fear level. Contrarily, those who visit the medical doctor only when needed and once a year were less likely to accept COVID-19 vaccine compared to participants who routinely visit the medical doctor.

**Conclusions:**

Our study showed a high level of acceptance of COVID-19 vaccination among Lebanese practicing dentists. And since knowledge about COVID-19 vaccination was associated with the vaccine acceptance, it should be improved and updated to further increase the acceptance rate. High acceptability of COVID-19 vaccination among dentists is expected to have a positive impact among the population in terms of increasing awareness and vaccine uptake.

## Background

An outbreak of pneumonia of unknown cause in Wuhan, China, was first reported in December 2019, and has raised intense concerns at an international level [1]. A novel coronavirus, severe acute respiratory coronavirus 2 (SARS-CoV-2), had been identified and isolated from patients in Wuhan by January 7, 2020, and has rapidly spread from Wuhan to the rest of the world [1]. On January 30, 2020, the Coronavirus Disease 2019 (COVID-19) outbreak has been declared as a “Public Health Emergency of International Concern” by the World Health Organization (WHO) [2]. On March 11, 2020, COVID-19 outbreak has been announced by the WHO as a global pandemic [2]. And up to March 25, 2021, there have been more than 123,000,000 confirmed cases with more than 2,710,000 deaths reported to the WHO worldwide. So far, no treatment for COVID-19 has been curative and universally approved, and in this regard, prevention remains the only crucial mean to fight against this virus which is constantly mutating into new variants, and among preventive measures, vaccination prevails [3].

At the time of this study, several COVID-19 vaccines have already been developed using different technologies, yet only mRNA vaccines (Moderna and Pfizer/BioNTech) with high efficacy (94–95%) and Janssen vaccine of Johnson and Johnson (one dose viral vector vaccine) have been authorized by the U.S. Food and Drug Administration (FDA) for emergency use [3–5]. COVID-19 vaccines produced by conventional methods (viral vectors, subunit particles, attenuated and inactivated viruses) have comparatively shown satisfactory but lower levels of efficacy ranging from 70 to 92% [3]. Currently, many countries have already started vaccination campaigns and Lebanon received its first batch of Pfizer/BioNTech COVID-19 vaccines on February 14, 2021; the vaccination campaign immediately started with elderly citizens and healthcare professionals (HCPs) including Lebanese dentists registered in the Lebanese Dental Association (LDA).

Oral healthcare professionals are at high risk of exposure to SARS-CoV-2 which spreads via respiratory droplets and aerosols resulting from oral surgical and dental procedures; cross-infection is the other (indirect) mode of transmission of this virus in the dental surgery [6–9]. Several measures and guidelines were adapted to clinical dental practice since the beginning of the outbreak to prevent transmissions among patients through cross-infection and transmission to and from the dentist himself/herself [7–9]. Nevertheless, the definitive solution to reduce the discussed risk, prevent further spread of the virus, and avoid complications associated with this disease, is to reach herd immunity, optimally through vaccination. Therefore, dental practitioners and their assistants are in utmost need to get vaccinated [10].

In reality, vaccines’ effectiveness depends on the race to vaccinate the world’s population before new resistant variants emerge, and this mainly relies on the acceptance of COVID-19 vaccination [3]. When a new vaccine is introduced, hesitancy usually arises against its safety and effectiveness. Since they are responsible to promote best health practices, HCPs’ influence on the public is crucial in spreading vaccination awareness [11–13]. Therefore, it is important to evaluate their COVID-19 vaccination acceptance and knowledge. Several researchers have evaluated such parameters among healthcare workers in few countries and showed an acceptance rate ranging from 27.7 to 85% among them [14–19], but, to our knowledge, no similar study was conducted in Lebanon yet. The current economic crisis in Lebanon has rapidly worsened due to the COVID-19 pandemic’s multiple lockdowns that hit hard the economy. At the time of this study, Lebanon was still far from acquiring herd immunity, which further threatened its recession, standard of living, and collapsing healthcare system, this issue being due to the unavailability of vaccines to the public yet and to the vaccine’s hesitancy in many social and occupational categories of Lebanese population. In addition, healthcare professionals’ COVID-19 vaccination acceptance is crucial in order to positively influence its general public acceptance and, consequently, reach herd immunity as soon as possible. And since vaccine’s acceptance is context-specific and varies with many factors, we aimed to assess the prevalence of COVID-19 vaccine acceptance among Lebanese dentists practicing in Lebanon, and to evaluate their knowledge towards COVID-19 vaccination. We also aimed to determine and analyze the predicting factors for the acceptance as many characteristics were collected from participants, among them, having comorbidities, frequency of medical visits, and the fact of living with medically vulnerable individuals which may seem to influence the vaccine acceptance.

## Methods

### Study design

This observational cross-sectional online survey was conducted in Lebanon between February 15 and February 22, 2021, in order to evaluate the acceptance of the COVID-19 vaccination among practicing dentists in Lebanon.

### Ethical considerations

Being observational and respecting participants’ anonymity and confidentiality, the Institutional Review Board (IRB) of the Lebanese University in Beirut–Lebanon waived the need for an ethical approval. Data were collected anonymously with no identifying or sensitive information. The allocated time for the questionnaire completion was around seven minutes.

### Study population and sample size calculation

Participants were eligible if they were Lebanese dentists practicing dentistry in Lebanon on a full or part-time basis and registered in the Lebanese Dental Association (LDA-Beirut or LDA-Tripoli).

Using the Epi Info™ software, and considering that around 5000 Lebanese dentists are currently practicing dentistry in Lebanon, the minimal sample size calculated was 357 with an expected acceptance prevalence of 50%, a confidence level of 95%, and an acceptable margin of error of 5%.

### Data collection

After reviewing literature [3, 10–13] and available information on COVID-19 vaccination from international guidelines [4, 5], a study instrument was developed in English and presented using the LimeSurvey® platform, an advanced online survey system. Expert opinions on the importance, intelligibility and clarity of the questionnaire’s content were considered before the final version was distributed. The questionnaire comprised four sections:

1. *Socio-demographic and work practice characteristics* including age, sex, marital status, living with vulnerable individuals, main area of practice, specialty(ies), years of experience, and professional affiliation(s), …
2. *Medical history and behavior section* including smoking status, frequency of medical visits, influenza and hepatitis B vaccinations, chronic medical conditions, allergies, experience with COVID-19 virus, fear related to getting infected with COVID-19 virus, and general attitude towards taking medications and the concept of vaccination. Participants were asked to indicate on a visual analogue scale from 0 to 10 how much they are scared of getting infected or reinfected with COVID-19 virus.
3. *COVID-19 vaccine acceptance section* including questions related to having already received COVID-19 vaccine (yes or no), willing to get vaccinated (yes, no, or undecided), and motivators and barriers to receiving the vaccine. COVID-19 vaccine acceptance is the main outcome dependent variable of this study that included the actual acceptance (having received the COVID-19 vaccine) and the willingness to get vaccinated if the participant had not yet received the vaccine.
4. *COVID-19 vaccine knowledge questionnaire* consisting of 13 true/false/don’t know questions aiming to evaluate the general knowledge of dentists on COVID-19 vaccines. This questionnaire included items on misconceptions on the potential vaccination risks.

### Procedure

Due to limitations in conducting face-to-face research during the current COVID-19 pandemic, the developed self-administered questionnaire was distributed online to Lebanese dentists. Invitations to participation were sent by the investigators via social networking platforms such as WhatsApp, Facebook, and Instagram using a snowball-sampling technique where participants were encouraged to pass on the questionnaire link to their colleagues. A short invitation text accompanied the survey link. Upon receiving and clicking the link, participants got automatically directed to the study aim and informed consent page, followed by the questionnaire to be filled.

### Statistical analysis

All data analyses were carried out using IBM SPSS Statistics for Windows (Version 26) (IBM Corp., Armonk, NY, USA). All tests were two-tailed and a *p*-value of less than 0.05 was considered statistically significant.

To describe the sample characteristics and to summarize variables, descriptive statistics were presented as frequency/percentage and mean ± standard deviation for categorical and continuous variables respectively. The main outcome of the study was the acceptance of COVID-19 vaccination.

For the knowledge index, a correct answer was given one (1) point and a wrong or “I don’t know” answer was given zero (0) point. The total knowledge index was calculated by summing all the answers and it ranged from 0 to 13, with a higher score indicating a better knowledge (Cronbach’s alpha: 0.662). A modified Bloom’s cut off point [20] was used to categorize the index into three levels of knowledge: high (80–100%; score range: 11–13), moderate (50–79%; score range: 7–10), and low (less than 50%; score range: 0–6). The fear of COVID-19 index was also categorized into three levels as per the previously mentioned modified Bloom’s cut off point.

To explore determinants of participants' acceptance of COVID-19 vaccination, bivariate analyses were first carried out between the dependent variable (acceptance) and the independent variables (chi-square test and Student t-test for categorical and continuous variables respectively), and those having a *p*-value ≤ 0.2 were then included in a multivariable modified Poisson regression model where adjusted prevalence ratio (aPR) values and their 95% confidence intervals (95% CI) were calculated. The model included originally the following variables: age, years of experience in dental practice, influenza vaccination during COVID-19 pandemic, frequency of medical visits, fear levels of getting infected or re-infected with COVID-19 virus, having chronic medical condition(s), COVID-19 vaccination knowledge levels, ever had COVID-19, and living with vulnerable individuals.

## Results

Out of 802 responses collected during the survey period, 253 were excluded due to incomplete data and 20 were also excluded for not meeting the inclusion criteria (being Lebanese and practicing dentistry on Lebanese territories). The remaining 529 participants were included in the study, and among them, responses regarding the knowledge section were missing in 20 included questionnaires. Baseline characteristics of the participants are shown in Table 1. The mean age of the 529 dentists was 40.54 ± 14.01 years, ranging from 22 to 78 years. There were 57.8% general practitioners and 42.2% specialists; participants were given the option to choose more than one specialty including Oral and/or Maxillofacial Surgery (9.6%), Orthodontics and Dentofacial Orthopedics (4.9%), Restorative and Esthetic dentistry (4.0%), Prosthodontics (6.2%), Endodontics (5.5%), Pediatric dentistry (4.2%), Oral Medicine (2.1%), Oral Pathology (2.1%), Oral and Maxillofacial Radiology (0.9%), and Implant dentistry (12.9%). Participant dentists had a mean experience of 16.1 ± 12.65 years, ranging from 0 to 50 years.

**Table 1:** General characteristics of the study population (N = 529)

Characteristics		Frequency (N)	Percentage (%)
*Sociodemographic and occupational characteristics*			
Age groups (years)	20–29	178	33.6
	30–39	101	19.1
	40–49	80	15.1
	50–59	111	21.0
	> 60	59	11.2
Sex	Males	292	55.2
	Females	237	44.8
Marital status	Single	197	37.2
	Married	315	59.5
	Divorced/Widowed	17	3.2
Living with^a^	Children	279	52.7
	Individuals older than 65 years	127	24.0
	Individuals with chronic diseases	124	23.4
	Non-applicable	114	21.6
Main area of dental practice	Northern Lebanon	66	12.5
	Eastern Lebanon	28	5.3
	Southern Lebanon	55	10.4
	Beirut	141	26.7
	Mount Lebanon	239	45.2
Specialty	General practitioner	306	57.8
	Specialist	223	42.2
Years of experience in dental practice	0–10	231	43.7
	11–20	104	19.7
	21–30	112	21.2
	31–40	74	14.0
	> 40	8	1.5
Affiliation	Private dental office and/or dispensary	416	78.6
	Hospital affiliation	20	3.8
	Academic affiliation	52	9.8
	University postgraduate student/resident	41	7.8
Practice outside Lebanon on a part-time basis	Yes	42	7.9
	No	487	92.1
*Health-related characteristics*			
Health coverage (medical insurance)	Insured	361	68.2
	Non-insured	168	31.8
Smoking status	Smoker	125	23.6
	Non-smoker	404	76.4
Frequency of medical visits	Routinely	53	10.0
	Once a year	140	26.5
	When needed	336	63.5
Drug consumption	I usually take when needed	275	52.0
	I only take when prescribed	171	32.3
	I don’t like to take medications	83	15.7
Having chronic medical condition(s)	Yes	80	15.1
	No	449	84.9
Influenza vaccination during COVID-19 pandemic	Vaccinated	157	29.7
	Non-vaccinated	372	70.3
Hepatitis B vaccination	Vaccinated	470	88.8
	Non-vaccinated	59	11.2
Drug allergy	Allergic	42	7.9
	Non-allergic	487	92.1
Other allergies (food, seasonal, latex, …)	Allergic	104	19.7
	Non-allergic	425	80.3
Vaccination concept opposition	Yes	38	7.2
	No	431	81.5
	Don’t know	60	11.3
*COVID-19-related characteristics*			
Ever had COVID-19	Yes	112	21.2
	No	417	78.8
Knowing someone hospitalized or dead from COVID-19	Yes	450	85.1
	No	79	14.9
Fear levels of getting infected or re-infected with COVID-19 virus	Low level (0–4)	118	22.3
	Moderate level (5–7)	223	42.2
	High level (8–10**)**	188	35.5
Encouraged family members to get the vaccine	Yes	461	87.1
	No	35	6.6
	Non-applicable	33	6.2
Already received or willing to receive COVID-19 vaccine (acceptance)	Yes	455	86.0
	No/undecided	74	14.0

^*^Add up to more than 100% due to multiple choice option

When participants were asked if they have ever received hepatitis B vaccine, 470 (88.8%) answered positively, among them only 20.2% were regularly checking their Hepatitis B surface antibody (anti-HBs) titers. Eighty (15.1%) participants had at least one medical comorbidity including arterial hypertension (56.3%), diabetes (30%), cardiac (22.5%), respiratory (11.3%), autoimmune (6.3%), thromboembolic (2.5%), liver (2.5%), kidney (1.3%) diseases, and/or cancer (5%).

Among participants who had COVID-19 (21.2%), 60 (53.6%) had mild, 44 (39.4%) moderate, and 8 (7.1%) severe symptoms. Only 26.8% out of 112 who had COVID-19 were aware of having acquired a significant level of antibodies; and 42% were still suffering from at least one symptom after recovery including fatigue (22.3%), shortness of breath (11.6%), olfaction disturbances (10.7%), cough (8%), mood swings (7.1%), muscle pain (7.1%), loss of gustation and/or headache (6.3%), depressive symptoms (5.4%), heart palpitations (4.5%), joint pain (3.6%), and/or chest pain (2.7%). Participant dentists reported a mean scale of fear from getting infected or re-infected with COVID-19 virus of 6.06 ± 2.92, ranging from 0 to 10. Participants who had already received the COVID-19 vaccine (10.8%) were asked to indicate the vaccine brand taken; 86%, 8.8%, and 5.3% have received Pfizer/BioNTech, Sinopharm, and Sputnik V vaccines, respectively. Participants who showed willingness to get vaccinated against COVID-19 (75.2%) were asked to indicate the vaccine brand(s) they feel comfortable to receive with a multiple-choice option: Pfizer/BioNTech (71.9%), Sputnik V (30.2%), Moderna (26.1%), AstraZeneca (21.4%), Janssen (12.3%), Sinopharm (12.1%), Sinovac (11.1%), and Novavax (2.1%).

**Fig. 1:**
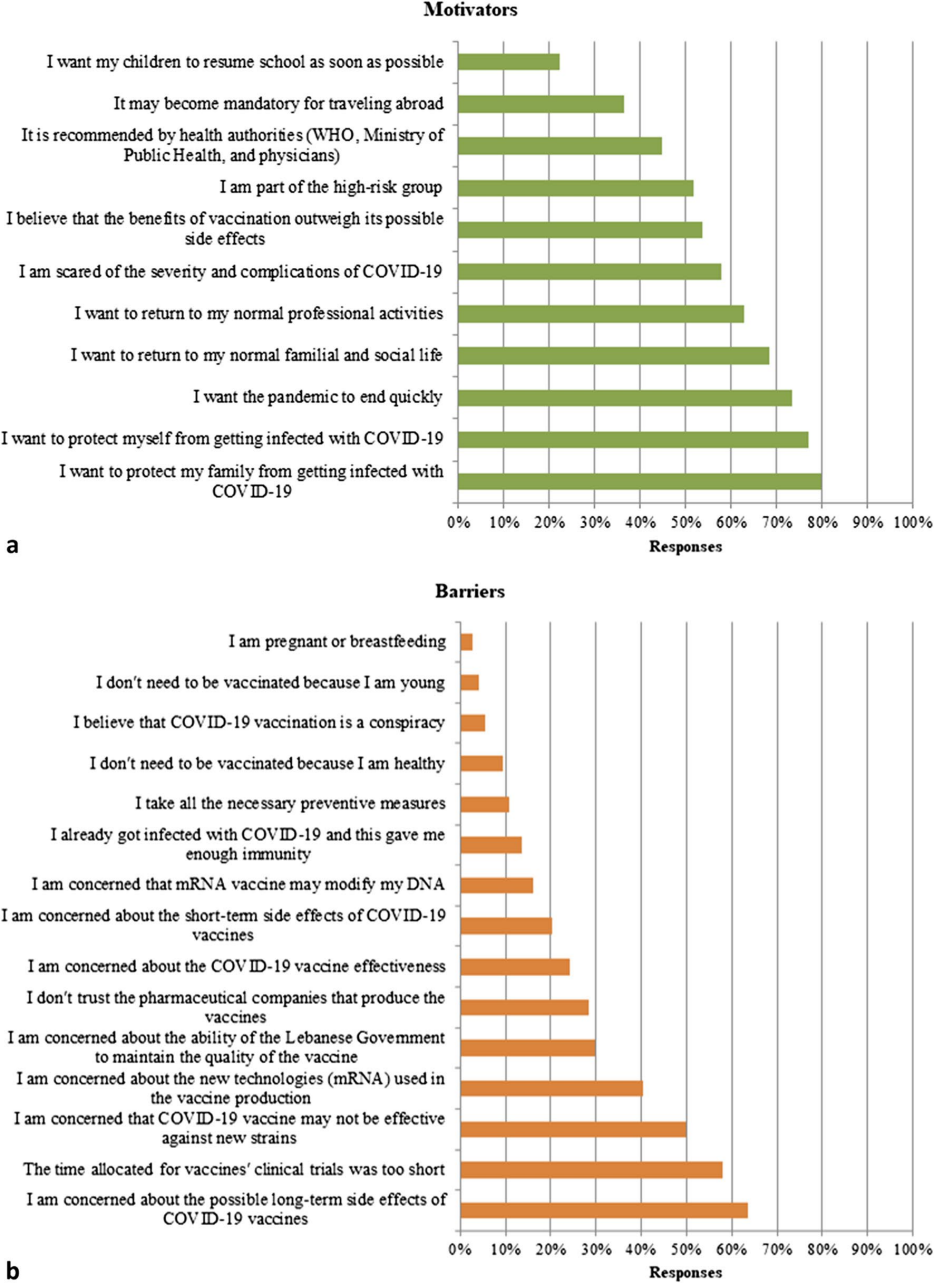
**a** Motivators and **b** barriers for accepting COVID-19 vaccination

The COVID-19 vaccination acceptance rate among participant dentists was 86%. Motivators for acceptance and barriers against it are shown in Fig. 1. Main motivators (Fig. 1a) for the vaccine acceptance were desire of the participants to protect their families from getting infected (79.8%), to protect themselves (76.9%), wanting the pandemic to end quickly (74.3%), and the desire to return to their normal social life (68.4%). Barriers (Fig. 1b) against getting vaccinated mainly included concerns about the possible long-term side effects of COVID-19 vaccines (63.5%), the rapidity of vaccines’ development (58.1%), and the effectiveness against new virus variants (50%). COVID-19 vaccination knowledge index ranged from 0 to 13 with a mean of 8.67 ± 2.45; knowledge levels and scores’ distribution are shown in Table 2.

**Table 2:** Dentists’ knowledge toward COVID-19 vaccination (N = 509)

		Frequency (N)	Percentage (%)
Knowledge levels	High (80–100%)	118	23.2
	Moderate (50–79%)	302	59.3
	Low (< 50%)	89	17.5

Knowledge items	Answers		
Q1-mRNA vaccine is a COVID-19 vaccine’s type	Correct	433	85.1
	Incorrect	76	14.9
Q2-Whole virus vaccine is a COVID-19 vaccine’s type	Correct	161	31.6
	Incorrect	348	68.4
Q3-Non-replicating viral vector is a COVID-19 vaccine’s type	Correct	190	37.3
	Incorrect	319	62.7
Q4-Protein subunit is a COVID-19 vaccine’s type	Correct	179	35.2
	Incorrect	330	64.8
Q5-COVID-19 vaccines decrease the risk of symptomatic infection with COVID-19 virus	Correct	422	82.9
	Incorrect	87	17.1
Q6-COVID-19 vaccines decrease the risk of transmission of COVID-19 virus	Correct	269	52.8
	Incorrect	240	47.2
Q7-Immunization is not acquired immediately after the COVID-19 vaccine injection	Correct	379	74.5
	Incorrect	130	25.5
Q8-All COVID-19 vaccines preparation techniques are new and were never used before	Correct	365	71.7
	Incorrect	144	28.3
Q9-Most of the confirmed side effects of COVID-19 vaccines are mild, resolving in 2–3 days	Correct	355	69.7
	Incorrect	154	30.3
Q10-People who were previously infected and have recovered from COVID-19 will never need to be vaccinated	Correct	461	90.6
	Incorrect	48	9.4
Q11-It is recommended to get the first and second shots of COVID-19 vaccine from two different manufacturers	Correct	423	83.1
	Incorrect	86	16.9
Q12-Preventive measures are no longer needed after vaccination against COVID-19	Correct	478	93.9
	Incorrect	31	6.1
Q13-Anyone can take the COVID-19 vaccine	Correct	298	58.5
	Incorrect	211	41.5

The results of the modified Poisson regression analysis for determinants of COVID-19 vaccine acceptance are shown in Table 3. Having received the influenza vaccine during the COVID-19 pandemic was linked to a 12% increase in the COVID-19 vaccination acceptance rate (aPR: 1.12; 95% CI: 1.06–1.20, *p* = 0.001). In addition, visiting the medical doctor only when needed (aPR: 0.87; 95% CI: 0.82–0.97, *p* = 0.007) and once a year (aPR: 0.89; 95% CI: 0.79–0.96, *p* = 0.004) were respectively associated with 13% and 11% decrease in the COVID-19 vaccine acceptance rate. Moreover, we found that having a moderate level of COVID-19 vaccination knowledge (aPR: 1.25; 95% CI: 1.09–1.44, *p* = 0.001) and having a high level (aPR: 1.30; 95% CI: 1.32–1.50, *p* < 0.001) were related to 25% and 30% increase in the vaccination acceptance rate; similarly, participants whose fear of COVID-19 level was high were significantly more likely to accept receiving a COVID-19 vaccine compared to those having a low fear level.

**Table 3:** Modified Poisson regression model showing predicting factors of COVID-19 vaccine acceptance

Variable	aPR*	95% CI**	*p*-value
*Influenza vaccination during COVID-19 pandemic*			
No (reference)	1	–	–
Yes	1.12	1.06–1.20	0.001
*Frequency of medical visits*			
Routinely (reference)	1	–	–
Once a year	0.89	0.79–0.96	0.004
When needed	0.87	0.82–0.97	0.007
*COVID-19 vaccination knowledge levels*			
Low (reference)	1	–	–
Moderate	1.25	1.09–1.44	0.001
High	1.30	1.32–1.50	< 0.001
*Fear levels of getting infected or re-infected with COVID-19 virus*			
Low (reference)	1	–	–
Moderate	1.07	0.96–1.19	0.209
High	1.11	1.002–1.227	0.045

Variables entered originally were: age, years of experience in dental practice, influenza vaccination during COVID-19 pandemic, frequency of medical visits, fear levels of getting infected or re-infected with COVID-19 virus, having chronic medical condition(s), COVID-19 vaccination knowledge levels, ever had COVID-19, and living with vulnerable individuals

^*^aPR = adjusted prevalence ratio

^**^95% CI = 95% confidence interval

## Discussion

As COVID-19-related morbidity and mortality are dramatically rising worldwide, prevention by vaccination is gaining ground among HCPs. Dentists are clinical specialists with a high risk of cross-infection in dental practice, and among preventive measures recommended in dentistry, vaccination remains the most prevailing one despite vaccine’s hesitancy, an emerging public challenge caused by rumors and misinformation [3, 6, 7].

Earlier studies have tackled the general population’s acceptance of COVID-19 vaccination [21–28] worldwide, and given the paucity of such studies in dental communities [18], we chose to conduct a survey on Lebanese dentists’ acceptance of COVID-19 vaccination.

Our acceptance rate result (86%) is similar to that found in a previous study [18] conducted on dental professionals (85%), and to that found in another study [19] conducted on healthcare workers that reported dentists’ acceptance rate of 82.5%. Studies on healthcare workers’ COVID-19 vaccine acceptance in Greece [19], France and French-speaking parts of Belgium and Canada [16], and China [15], also showed comparable acceptance rates of 78.5%, 76.4% and 72.4% respectively. These findings are substantially higher in comparison to a study conducted on Congolese healthcare workers [14], where an acceptance rate of 27.7% was found; this low rate could be explained by the fact that the survey was conducted at a time where full scientific information on COVID-19 were not yet elucidated and vaccines’ clinical trials not yet launched, and according to authors, this relatively low rate may also be explained by the harm of social networks and spread of misinformation [14]. Another study [17] showing a low acceptance rate was conducted among healthcare workers in the USA: only 36% of respondents were willing to take the vaccine as soon as it becomes available, whereas 56% were hesitant of taking it or would prefer to wait for new data regarding vaccination. This study that classified healthcare workers into four major categories showed that direct medical providers had higher vaccine acceptance (49%) compared to administrative staff (34%), and direct patient care providers had the lowest vaccine acceptance rate (27%) with nearly two-thirds of them (62%) choosing to postpone their vaccination after reviewing its safety data [17].

Studies targeting the general population in different countries reported COVID-19 vaccine acceptance rates of 93.3%, 91.3%, 67%, 64.7%, 53.1%, 37.2% and 36.8%, in Indonesia, China, United States, Saudi Arabia, Kuwait, Hong Kong, and Jordan respectively [21–26, 28], whereas a global survey has found an acceptance rate of 71.5% [27].

Our results showed a high rate of acceptance which may be explained by several factors. Being part of the high-risk group of getting COVID-19, and the exclusive availability of Pfizer/BioNTech mRNA COVID-19 vaccine in Lebanon at the time of the study (which proved to have a high efficacy and safety), are possible reasons that have positively influenced Lebanese dentists’ COVID-19 vaccination acceptance. This high rate may also be justified by the number of deaths due to COVID-19 in Lebanon which exceeded 5000 at the period of the survey (among them many dentists and physicians), therefore possibly influencing the fear level which, in turn, have positively affected the acceptance rate. Other potential influencing considerations included the extreme economical and financial crises that were exacerbated by the multiple total lockdowns in Lebanon, rendering the citizens desperate to return to normal professional activities. Key motivating factors for COVID-19 vaccination acceptance among dentists and barriers against it, complied with findings obtained in previous studies [17, 21–23, 25, 28]. The overbalance of motivators on the scientifically-unproven barriers against vaccination possibly explains the high acceptance rate obtained in our study.

Our multivariable analysis showed that the frequency of medical visits of practicing dentists is a potential predictor for COVID-19 vaccine acceptance, a finding similar to that of a Lebanese study [29] assessing regular influenza vaccination, where participants who visit their physician only when needed were found to be less likely to get vaccinated [29]. This finding could be explained by the fact that a health-conscious person would regularly visit her/his treating physician in order to remain healthy, and therefore, is more likely to get vaccinated against any life-threatening disease. Vaccination against influenza during the pandemic was found to be another positive predictor for COVID-19 vaccine acceptance, which complies with the results found in the French and French-speaking parts of Belgium and Canada study [16] where participants previously vaccinated against influenza were less likely to have COVID-19 vaccine hesitancy. Our finding was also similar to that of the Chinese study [22] which reported that participants who got vaccinated for influenza in the past season had greater odds of accepting COVID-19 vaccine. The study conducted in Kuwait [25] has shown that influenza vaccination was a predictor for COVID-19 vaccine acceptance as well. At the beginning of the COVID-19 pandemic, it was thought that influenza vaccination would possibly protect against COVID-19; this have possibly encouraged people who are already predisposed to accept the COVID-19 vaccination concept or any other vaccination, to receive the flu vaccine.

On the other hand, we have found in the present study that COVID-19 vaccination knowledge and fear of getting infected with SARS-CoV-2 were associated with vaccine acceptance among Lebanese dentists. Dentists who are knowledgeable about COVID-19 vaccination such as safety, mechanism of action, … had much less hesitancy to receive the vaccine, and this explains the positive predicting influence on the acceptance. Moreover, since vaccination is the most predictable mean to prevent infection and stop the pandemic, fearful individuals are more likely to seek vaccination in order to avoid contracting the disease.

To our knowledge, this is the first study conducted in Lebanon assessing COVID-19 vaccination acceptance among healthcare professionals and specifically, among dentists. In addition, our sample size was large enough to carry out a multivariable analysis that allowed exploring an important number of potential determinants of vaccine acceptance while controlling for confounders. Our study had some limitations. One was using the non-probability snowball sampling technique which may have generated a selection bias and may therefore have compromised the generalizability of our results. Unfortunately, it was not possible to compare participant dentists’ characteristics to those of the target population due to the lack of data on the characteristics of the whole population of dentists in Lebanon. In addition, online-surveying method, which is restricted to computerized and internet-friendly users, had possibly created a selection bias as well, especially that those who have access to social media and internet may have better knowledge regarding COVID-19 vaccination information and increased health literacy. Furthermore, prevalence of vaccine acceptance could be overestimated because dentists not interested in getting vaccinated were possibly not motivated to participate in this survey. Also, and because our study was conducted in a period during which Lebanese dentists have started to get vaccinated, encouragements between colleagues for receiving vaccination may have had a positive impact on raising acceptability rate.

## Conclusions

In the attempts to control COVID-19 pandemic, hopes are put on vaccination as the most effective preventive solution so far. Our study demonstrated a high level of acceptance of COVID-19 vaccination in Lebanese practicing dentists. Since dentists are among primary healthcare providers, such acceptance is critical and its implications are not limited to the dental professionals themselves, but may exert influence on the community and public acceptance and awareness. Together with other healthcare professionals, the duty to promote recommended public health measures against COVID-19 is a timely and difficult challenge. High acceptability of COVID-19 vaccination among dentists is expected to have a positive impact among the population in terms of increasing awareness and vaccine uptake. After all, and since knowledge about COVID-19 vaccination was found to be associated with the vaccine’s acceptance, it should be improved and updated in order to further increase the acceptance rate.

Further studies are warranted to evaluate the actual vaccination rate and overall impact on dental healthcare system after the end of the vaccination campaign.

## Data Availability

The datasets used and/or analyzed during the current study are available from the corresponding author on reasonable request.

## Abbreviations

aPR: Adjusted prevalence ratio
COVID-19: Coronavirus disease 2019
FDA: U.S. Food and drug administration
HCP: Healthcare professional
HCW: Healthcare worker
SARS-CoV-2: Severe acute respiratory coronavirus 2
